# Comparative Pathogenesis of Cancers in Animals and Humans

**DOI:** 10.3390/vetsci3030024

**Published:** 2016-09-13

**Authors:** Jaime F. Modiano

**Affiliations:** 1 Animal Cancer Care and Research Program, University of Minnesota, St. Paul, MN 55108, USA; modiano@umn.edu; Tel.: +1-612-625-7436; 2 Department of Veterinary Clinical Sciences, College of Veterinary Medicine, University of Minnesota, St. Paul, MN 55108, USA; 3 Masonic Cancer Center, University of Minnesota, Minneapolis, MN 55455, USA; 4 Center for Immunology, University of Minnesota, Minneapolis, MN 55455, USA; 5 Stem Cell Institute, University of Minnesota, Minneapolis, MN 55455, USA; 6 Department of Laboratory Medicine and Pathology, School of Medicine, University of Minnesota, Minneapolis, MN 55455, USA

## Editorial

As Guest Editor of *Veterinary Sciences*, I am honored to introduce the special issue, “Comparative Pathogenesis of Cancers in Animals and Humans”. For this issue, we assembled a timely and informative collection of 18 review manuscripts describing the current state-of-the-art for oncological sciences in companion animals, and specifically, how this informs and enhances our understanding of human cancer. As you read each article, I urge you to keep the following two questions in mind: (1) When is a model a model? And, (2) Is it fair to say that cancer is a result of our evolutionary legacy?

Merriam Webster defines an animal model as, “an animal sufficiently like humans in its anatomy, physiology, or response to a pathogen to be used in medical research in order to obtain results that can be extrapolated to human medicine”; or “a pathological or physiological condition that occurs in such an animal and is similar to one occurring in humans”. Superficially, the resemblance of cancer (and other diseases) of companion animals to like conditions in humans led Calvin Schwabe to propose the concept of One Medicine (now called One Health) in his book, Veterinary Medicine and Human Health in 1964 [1]. This was a natural sequel to Virchow’s maxim a century earlier stating that, “between animal and human medicine there is no dividing line—nor should there be. The object is different but the experience obtained constitutes the basis of all medicine”.

In the period between the early 1960s and the late 1980s, there was a surge in veterinary epidemiology and public health studies, which coincided with changing demographics and the status of pets in our society. Epidemiological studies done during this time period identified hematologic malignancies (principally lymphomas), skin cancers, and sarcomas as highly prevalent in dogs. This also was a high point for studies in viral oncology, especially in cats, on the heels of the discovery of FeLV and other viruses. Recent estimates that indicate the lifetime risk for cancer in dogs, cats, and humans is similar have further entrenched the assumption that observations regarding epidemiology and cancer biology in companion animals can be directly extrapolated to humans, and vice-versa. 

However, is this true? Do shared features of morphology and topology between companion animal and human cancers make them equivalent diseases? To begin to answer this, it is worth considering the second question I posed above. There is inevitable, inherent bias in studies of cancer causation. Strong evidence in one area of study can unduly influence hypotheses and interpretations in other, unrelated areas of study. For example, there are undeniable, causal links between habitual tobacco use, or exposure to mutagens and human lung, bladder and blood cancers; between exposure to ultraviolet radiation and human skin cancer; and between infections with certain viruses or bacteria and some human blood-derived cancers and solid cancers. These etiologies account for a large proportion of individual human cancer cases across the world, but they do not explain many other types of common or rare cancers. And still, there is a disproportionate emphasis on identifying environmental causes for virtually all human-cancers. 

Witness the visceral reactions after Tomasetti and Vogelstein published, “*Variation in cancer risk among tissues can be explained by the number of stem cell divisions*” [2]. Tomasetti and Vogelstein suggested that, “only a third of the variation in cancer risk among tissues is attributable to environmental factors or inherited predispositions.” In other words, they proposed that up to two thirds of human cancers might be due to “bad luck,” or random mutations arising during the process of DNA replication in normal stem cells. This intriguing paper was counterintuitive to standing dogma, and it drew overwhelming negative responses. For example, Ashford et al. called the report “dangerously misleading” [3] and other readers questioned both their methodology and their conclusions. It remains with each of us to carefully and objectively analyze the data and reach cautiously supportable conclusions.

We must use the same approach as we view the utility of domestic animals as “models” for human disease, and even for animal disease. Over the past four or five decades, common cancers of dogs, and in particular lymphoma, osteosarcoma, and melanoma (I refer to these as “the big three”), have been advanced as reliable and clinically relevant models of human disease. Much of the evidence comes from observations on the natural history of these tumors and the superficial resemblance between the canine and the human versions of these diseases with regard to anatomy, topology, patterns of metastasis and response to therapy. Nonetheless, we must continue to question how, when, and where these diseases can truly be used to model human disease. The manuscripts by Seelig et al. [4], Varshney et al. [5], Fan and Khanna [6], and Tomoko-Nishiya et al. [7] can help us start navigating that path. To quote Seelig, “In both species diffuse large B-cell lymphoma (DLBCL) is the most common subtype (of lymphoma), but this is where the similarities end.” Their article, like all the others in this collection, is heavily cited to provide the reader abundant background material and context. Back-to-back articles by Tomiyasu and Tsujimoto [8] and by Zandvliet and Teske [9] offer a glimpse into mechanisms of drug resistance with emphasis on lymphoma.

Farruk et al. [10] tackle the challenging topic of mammary cancer. The authors deftly cover abnormalities in cell cycle and microRNAs that have been associated with canine mammary cancer and with human breast cancer. Kennedy et al. [11], Kim et al. [12], and Dickerson and Bryan [13] address the biology and genetics of histiocytic sarcomas and vascular sarcomas. The papers by Kennedy and Kim focus on diagnostic challenges and the evolution in our understanding of the cells of origin for these aggressive mesenchymal tumors that are common in dogs, but rare in humans. Dickerson and Bryan review the role of beta-adrenergic signaling in sarcomas and propose that modulating these pathways could provide therapeutic traction for these and other incurable cancers.

The manuscript by Mochizuki and Breen on BRAF mutations [14] is especially noteworthy because it addresses the concept of molecular vs. histologic homology. Mochizuki and Breen describe single amino acid substitutions (V595E) in the BRAF signaling domain in canine urothelial tumors. These are homologous to the single amino acid substitutions (V600E) that are present in the BRAF signaling domain in 25%–50% of human melanomas. Both mutations lead to constitutive activation of MAPK pathways, presumably contributing to uncontrolled proliferation. This is the second such instance documenting recurrent and highly prevalent homologous mutations in dissimilar tumors of dogs and humans (the first was a tandem duplication of the KIT juxtamembrane domain in canine mast cell tumors and human gastrointestinal stromal tumors). While skin melanocytes and urothelial lining cells have different ontogenesis and reside in different microenvironments, the context in which the mutations occur could teach us about the cellular response to DNA damaging agents. Furthermore, if BRAF mutations were proven to be drivers of canine urothelial cancers, it would create therapeutic opportunities for a disease where few exist. And furthermore, the canine condition also would provide opportunities for ethical testing of novel BRAF targeted therapies.

The review by Dervisis and Klahn [15] offers contemporary insights into one of the most active areas in cancer therapy, namely tyrosine kinase inhibition. They approach their subject in a refreshingly honest manner, emphasizing the almost inevitable problem of resistance and clearly identifying unanswered questions in the field for both human and companion animal cancer patients.

Tandem articles by Anderson and Modiano [16] and by Regan and Dow [17] describe recent advances in cancer immunotherapy for companion animals and how this knowledge might be used to improve immunotherapy for humans. The former paper emphasizes adaptive immunotherapy, describing several approaches that are at or near clinical translation in the veterinary clinic for dogs. The latter paper describes methods for manipulating the innate immune system to control tumor growth and metastasis, also denoting therapies that have been tested in canine cancer patients.

It might be safe to call feline oncology an “emerging field”. Cannon [18] expertly guides the reader through common feline cancers that create distinct and complementary models to those described for dogs, including feline oral squamous cell carcinoma, mammary gland tumors, and injection site sarcomas. These three tumors all seem to have different natural histories than their canine counterparts, and so they offer potential “models” for specific subtypes of the human conditions, which Cannon underscores by having each disease neatly paired with a potential molecular or targeted therapy approach. Thomas [19] elegantly describes exciting advances in cytogenomics of feline injection site sarcomas, lymphomas, and mammary gland tumors that were made possible by the recent sequencing and assembly of the feline genome. The knowledge gained from these studies could guide future therapeutic modeling.

Finally, Mazcko and Thomas [20] review the process through which the Pfizer-Canine Comparative Oncology and Genomics Consortium (CCOGC) biospecimen repository was established, as well as the processes of quality assurance and quality control that are needed to ensure robustness of such a resource.

Overall, the articles in this collection are complementary and diverse. Key opinion leaders in the field have authored a collection that offers a meaningful resource for the cancer expert and the cancer novice alike, and the satisfying redundancy among the articles provides confidence of veracity. When I agreed to edit this issue, I sought to “build a contemporary collection to serve the needs of the community for time to come, and where we could reach the bounds of imagination supported by hard science.” Thanks to the hard work of the authors and the editors, I believe we achieved our goal in spectacular form.
